# Expression Profiles of Co-Inhibitory Receptors in Non-Urothelial Bladder Cancer: Preclinical Evidence for the Next Generation of Immune Checkpoint Inhibitors

**DOI:** 10.3390/cancers17132210

**Published:** 2025-07-01

**Authors:** Severin Rodler, Stephan T. Ledderose, Raphaela Waidelich, Jakob Kohler, Andrea Sendelhofert, Jozefina Casuscelli, Gerald Schulz, Christian G. Stief, Lennert Eismann

**Affiliations:** 1 Department of Urology, University Hospital of Ludwig-Maximilian-University, 81377 Munich, Germany; 2 Department of Urology, University Hospital Schleswig-Holstein, Campus Kiel, 24105 Kiel, Germany; jakob.kohler@uksh.de; 3 Institute of Pathology, Ludwig-Maximilian-University, 80337 Munich, Germany

**Keywords:** variant histology, bladder cancer, squamous-cell carcinoma, adenocarcinoma, coinhibitory receptor, TIM-3, LAG-3, TIGIT

## Abstract

Immune checkpoint inhibition is a cornerstone of bladder cancer therapy, but its efficacy in non-urothelial subtypes of bladder cancer is limited, and the prognosis remains poor. Therefore, we investigated the potential of the immune checkpoint molecules TIM-3, TIGIT, and LAG-3 in squamous-cell carcinoma (SCC) and adenocarcinoma (ADENO) of the urinary bladder. Tumor-infiltrating lymphocytes (TILs) showed a high expression of TIM-3 and TIGIT in both SCC and ADENO, while LAG-3-positive TILs were absent in ADENO and present in 46% of SCC. Quantitative analysis revealed age-independent expression of TIM-3 in SCC (r = −0.001, *p* = 0.997) and ADENO (r = 0.135, *p* = 0.549), with increasing age correlating with higher expression of TIGIT (r = 0.157, *p* = 0.242) and LAG-3 (0.106, *p* = 0.436) in the SCC cohort and of TIGIT (r = 0.276, *p* = 0.214) in the ADENO cohort. Male patients showed increased TIGIT scores in ADENO (*p* < 0.01). Of note, a high infiltration of TIM-3-TILs (*p* = 0.048) correlated with worse progression-free survival in SCC. These results highlight the differential expression of co-inhibitory receptors in non-urothelial bladder cancer subtypes and provide preclinical evidence for new therapeutic targets. Biomarker testing prior to clinical trials is essential for identifying the most suitable patients for targeted immunotherapy.

## Simple Summary

This study examines the expression patterns of new immune co-receptors in variant histology bladder cancer. TIM-3, TIGIT, and LAG-3 show differential expression in squamous-cell and adenocarcinoma, varying by age and gender. Biomarker testing is essential for future trial success.

## 1. Introduction

Immune checkpoint inhibitor (ICI) therapy is a standard treatment for conventional urothelial carcinoma alongside platinum-based chemotherapy and, more recently, antibody–drug conjugates (ADCs). Variant histologies (VHs) represent 25% of all bladder cancer and are characterized by poor prognosis and limited response rates to immunotherapy [1,2]. In the past, VHs have been treated analogously to conventional urothelial bladder cancer with limited success. However, ongoing research has unveiled that each specific histological subtype presents a unique molecular genetic profile and clinical behavior [3,4]. Given that most VHs are diagnosed at an advanced local stage or are already present with metastatic disease, systemic therapy constitutes the cornerstone of treatment [5]. Small retrospective cohort studies suggest a survival benefit using combination chemotherapy in non-urothelial carcinoma of the bladder; however, overall survival in the metastatic stage is limited to 13 months [6,7]. The use of chemotherapy in a neoadjuvant setting has shown varying results. Patients with adenocarcinoma (ADENO) demonstrated survival benefits, whereas squamous cell-carcinoma (SCC) is considered thermoresistant [8]. In that background, the PURE-1 trial investigated prembrolizumab monotherapy as neoadjuvant regimens and investigated patients with VHs. In total, 19 patients with predominant VHs were included, and interestingly, SCC showed response in 86%, but no response was seen in all other subtypes [9]. Preliminary results of a single-arm phase II trial investigating neoadjuvant pembrolizumab with methotrexate, vinblastine, doxorubicin, and cisplatin have shown pathological response in 9 out of 17 patients (53%). Ten patients experienced grade 3 or higher adverse events, reflecting the high toxicity of this multidrug combination therapy [10]. A promising mouse model has shown that targeting additional immune checkpoints might reduce treatment resistance to conventional anti-PD-1 and chemotherapy [11]. Therefore, the clinically evolving landscape of oncological research urges exploration beyond immune checkpoint monotherapies towards synergistic approaches using combination immunotherapies to enhance treatment efficacy [12]. While PD-1/PD-L1 inhibitors remain central to current immunotherapeutic strategies, as seen in the PUR-1 trial, their limited efficacy in specific VHs or the relevant number of patients demonstrating no response even to high-toxicity combination therapies highlights the urgent need to identify additional immune checkpoints involved in resistance mechanisms [9]. Central to this endeavor is deciphering the cancer-immunity cycle in VHs, and obtaining insights into the expression patterns of key molecules is crucial for tailoring future trials to enhance response rates to next-generation ICI [13].

Recent advancements have identified co-inhibitory receptors like T-cell immunoglobulin and mucin domain-containing protein-3 (TIM-3), lymphocyte activation gene-3 (LAG-3), and T-cell immunoreceptor with immunoglobulin and ITIM domain (TIGIT) as promising targets for novel checkpoint inhibition [12]. These molecules are known to mediate T cell exhaustion and contribute to immune escape, particularly in tumors with low PD-L1 expression or resistance to PD-1/PD-L1 blockade. In this pursuit to further understand the immunogenic landscape of adenocarcinoma (ADENO) and squamous-cell carcinoma (SCC), the most relevant non-urothelial VHs of bladder cancer, we analyzed quantitative expression patterns and coreceptor distribution for relevant clinical parameters and tumor characteristics to identify patients suitable for future trials.

## 2. Methods

This study was approved by the ethics committee of Ludwig-Maximilian University Munich (Ref. 20-179) prior to initiation. Patients who underwent radical cystectomy (RC) for muscle-invasive bladder cancer (MIBC) between 2004 and 2019 were retrospectively analyzed. Histopathological review was performed by experienced genitourinary pathologists at our institution, identifying cases of pure squamous-cell carcinoma (SCC) and adenocarcinoma (ADENO) of the bladder. Pathological staging followed the AJCC/UICC TNM classification (8th edition) and WHO 2016 criteria [14].

Patients underwent RC following a standardized protocol that involved lymph node dissection and urinary diversion, performed either as an ileal neobladder or an ileal conduit. Postoperative recovery was enhanced following Enhanced Recovery After Surgery (ERAS) protocols after RC. Rehabilitation was offered to all patients by the institutional discharge management. Psychooncological counseling was offered during the hospital stay.

Follow-up was performed according to the European guidelines at either our institution or at office-based urologists. For receiving clinical follow-up data, patients were included in a prospective registry. After providing informed consent for retrieving clinical data and being contacted after the hospital stay, patients were followed up after 3 and 6 months and yearly thereafter via mail. The patient registry is generated and edited by dedicated data managers. Data is checked prior to entry for plausibility.

Tissue microarrays (TMAs) were constructed from formalin-fixed, paraffin-embedded (FFPE) tumor blocks using 1 mm punch biopsies from three distinct tumor regions per case. Sections (4 µm) were cut and subjected to immunohistochemistry (IHC) for TIM-3, TIGIT, and LAG-3. Antigen retrieval was performed using standardized heat-induced protocols (Target Retrieval Solution [Agilent] or Novocastra [Leica], depending on antibody), followed by incubation with primary monoclonal rabbit antibodies (TIM-3: 1:80, Cell Signaling D5D5R; TIGIT: 1:150, Cell Signaling E5Y1W; LAG-3: 1:600, Abcam EPR4392(2)). Detection was achieved via polymer-based systems (ImmPRESS or ZytoChem Plus) and visualized using either DAB+ or Permanent AP Red chromogens. Counterstaining was performed with hematoxylin, and tonsil tissue served as a positive control in each run.

Immunoreactive tumor-infiltrating lymphocytes (TILs) were quantified by manual cell counting at 400× magnification across at least two representative fields. The mean cell count per tumor was calculated, and cases with core loss or lacking tumor tissue were excluded. For further analysis, biomarker expression was dichotomized into low vs. high infiltration based on median cut-off values: SCC—LAG-3 (≥1), TIGIT (≥11), TIM-3 (≥14); ADENO—TIGIT (≥11), TIM-3 (≥1); LAG-3 was not evaluable in ADENO due to insufficient representation.

Continuous variables were displayed as medians with interquartile ranges. Statistical analyses included the Kruskal–Wallis test for comparisons by sex and T-stage. Pearson correlation and scatter plots were used to display and analyze age association. The Kaplan–Meier method with log-rank tests was used to calculate overall survival (OS) and progression-free survival (PFS) in this patient cohort. A *p*-value < 0.05 was considered statistically significant.

## 3. Results

In total, 83 patients with SCC (n = 59) and ADENO (n = 24) met the inclusion criteria and were included for further analysis. The median age of patients was 68 (IQR 59–77), and the median follow-up was 46 months. 36 (43.4%) patients were female, and 71 (85.5%) patients presented with at least T3 disease at the time of RC. Baseline characteristics were equally distributed between the two groups. Median OS was 21 months for SCC and 27 months for ADENO (*p* = 0.877). In line with this, the median PFS was 17 months in the SCC cohort and 17 months in the ADENO cohort (*p* = 0.603).

### 3.1. Expression Patterns

Expression of TIM-3 and LAG-3 on tumor-infiltrating lymphocytes (TILs) was significantly higher in SCC (median Score_TIM-3_: 14, median Score_LAG-3_: 1) compared to ADENO (median Score_TIM-3_: 1, median Score_LAG-3_: 0; *p* < 0.001 for both VH). Notably, there were no LAG-3-positive TILs in ADENO. TIGIT scores were similar in SCC (median Score_TIGIT_: 11) and ADENO (median Score_TIM-3_: 14, *p* = 0.9, see Table 1, Supplementary Figure S1).

**Figure 1:**
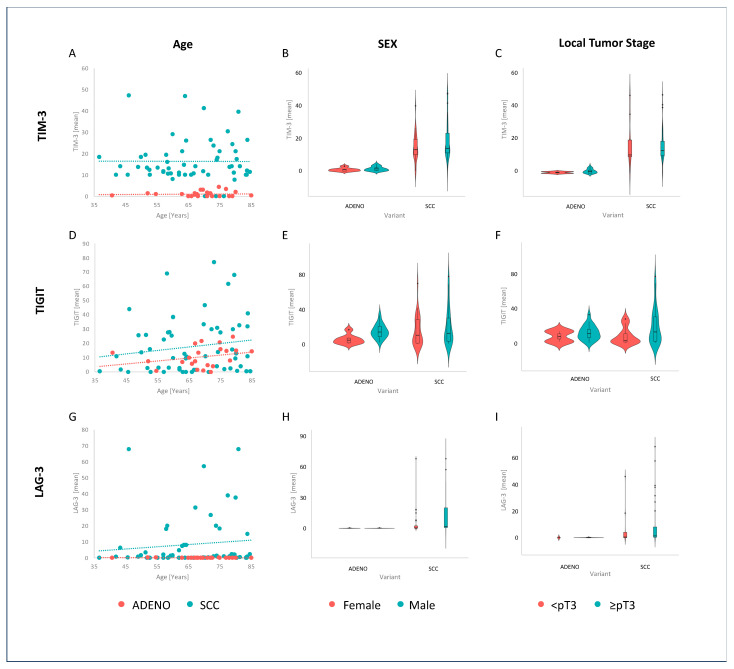
Impact of age (**A**,**D**,**G**), sex (**B**,**E**,**H**), and local tumor stage (**C**,**F**,**I**) on expression patterns of coinhibitory receptors (TIM-3: (**A**–**C**); TIGIT: (**D**–**F**); LAG-3: (**G**–**I**)) in variant histologies. Red indicates ADENO samples and blue SCC samples in (**A**,**D**,**G**). Red violin plots indicate female and blue male samples in (**B**,**E**,**H**). In (**C**,**F**,**I**), red violin plots indicate <pT3 samples whereas ≥pT3 samples are marked in blue.

In ADENO, TIM-3-positive TILs were constantly distributed over different ages (r = −0.07, *p* = 0.557), whereas there was a tendency of increasing TIGIT-positive TILs in older patients (r = 0.14, *p* = 0.218). Regarding the analysis for sex-specific differences, TILs showed no difference in TIM-3 expression between sexes either in SCC (*p* = 0.3) or ADENO samples (*p* = 0.25). For TIGIT, we detected significantly higher TIGIT expressions in male ADENO patients (*p* = 0.0069); in contrast, TIGIT expression showed no difference between sexes in SCC (*p* = 0.2). Interestingly, male patients with SCC tended to have higher LAG-3 scores (*p* = 0.054). Next, we compared localized disease (<pT3) and locally advanced disease (≥pT3) for all three co-inhibitory receptors. We could not demonstrate any difference for TIM-3, TIGIT, and LAG-3 in SCC (*p* = 0.58; *p* = 0.095; *p* = 0.38) and ADENO (*p* = 0.34; *p* = 0.5; *p* = 0.58). For further details, refer to Figure 1.

### 3.2. Prognostic Value

**Figure 2:**
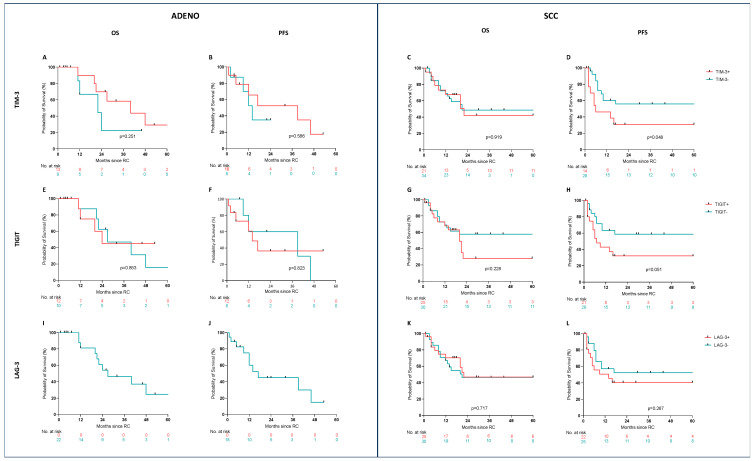
Prognostic value for overall and progression-free survival of TIM-3, LAG-3, and TIGIT. Prognostic value of inhibitory coreceptor expression was analyzed for overall survival (OS) and progression-free survival (PFS) for adenocarcinoma (ADENO—(**A**,**B**,**E**,**F**,**I**,**J**)) and squamous-cell carcinoma (SCC—(**C**,**D**,**G**,**H**,**K**,**L**)). The median expression of the respective coreceptor was used as a cut-off. Abbr.: ADENO: adenocarcinoma, SCC: squamous-cell carcinoma, OS: overall survival, PFS: progression-free survival, RC: radical cystectomy.

Next, we analyzed the predictive value of the three co-inhibitory receptors. Patients were categorized in a low and high expression group based on the median expression of the entire cohort (SCC: LAG-3 (≥1); TIGIT (≥11); TIM-3 (≥14). PFS was significantly shorter for TIM3^high^ (*p* = 0.048) in SCC patients. Co-inhibitory receptor expression did neither in SCC nor in ADENO significantly affect overall survival (see Figure 2).

## 4. Discussion

This study provides a comprehensive analysis of the intricate immune profiles of three relevant co-receptors—TIM-3, LAG-3, and TIGIT—in the most relevant non-urothelial subtypes of bladder cancer. Patients with ADENO and SCC of the bladder frequently present at advanced tumor stages and are particularly vulnerable to receiving ineffective therapies due to a lack of evidence-based treatment regimens. The unique, aggressive biological behavior challenges clinicians, and further thorough understanding of the tumor microenvironment is needed for novel treatment targets beyond the landscape of conventional PD-1/PD-L1. Potential next-generation immune checkpoints specific to ADENO and SCC of the bladder are discussed, with exploration across various clinical parameters aimed at identifying suitable candidates for forthcoming trials.

Variant histologies (VH) of bladder cancer remain associated with limited life expectancy, even among patients initially treated with curative intent. However, survival outcomes have been subject to ongoing debate, particularly whether they differ from pure urothelial carcinoma. Moschini et al. observed that pure but not mixed VHs of bladder cancer are associated with poor survival compared to pure urothelial carcinoma [15]. In line, SCC reveals inferior survival to pure urothelial carcinoma in a study by Agrawal et al. [16]. In our cohort, the median OS for SCC was 21 months. While being less frequent than SCC, ADENO of the bladder revealed poor outcomes with a 2-year OS of 54.8% [17,18]. Compared to pure urothelial carcinoma, ADENO has worse outcomes across different treatment settings but is positively impacted by RC [19]. In line with the expected biological behavior of ADENO, the median OS for ADENO was 27 months in our cohort. Part of this biological behavior of both VHs contributing to short survival times is that both histologies present at advanced stages [20]. As expected, patients in our cohort presented in 93% of all cases for ADENO and 82% for SCC with locally advanced disease.

Gender-based disparities in bladder cancer incidence and outcomes are well documented. Conventional bladder cancer is predominantly found in men, but advanced disease is more common in women [21,22]. For SCC, prior studies have suggested a more equal sex distribution [23], while ADENO has been shown to predominantly affect males (male 63% vs. female 37%) [18]. Our findings corroborate these reports, with ADENO exhibiting a male predominance (70% male vs. 30% female) and SCC showing a near-equal gender distribution (51% male vs. 49% female). These observations warrant further exploration into sex-based immunogenomic differences, as they may influence not only disease behavior but also response to immune checkpoint inhibition.

Notably, our findings highlight significantly elevated TIM-3-positive TILs in ADENO and SCC. Antibodies targeting TIM-3, which have been clinically evaluated, are now the focal point of ongoing research, particularly in tumors evading current immunotherapies that solely target PD-1 [24]. Furthermore, TIM-3 demonstrated consistent expression across all age groups and exhibited no significant variations across genders or tumor stages. Thus, TIM-3 emerges as a universal therapeutic target applicable to all patients and warrants evaluation in local and advanced tumor stages of ADENO and SCC.

In our cohort, LAG-3 expression was absent in ADENO tumors, whereas 46% of SCC cases exhibited a high expression profile, with a positive correlation observed between increasing age and LAG-3 expression. Given the lack of effective systemic therapies reported for SCC to date, the combination blockade of PD-1 and LAG-3 holds promise, particularly among older patients ineligible for other treatment options. Tebotelimab, a bispecific antibody targeting PD-1 and LAG-3, has been investigated in solid tumors but has yet to be explored in bladder cancer [25].

TIGIT showed comparable expression levels in ADENO and SCC, with a tendency for higher expression among older patients. Sex-based differences in TIGIT expression were noted in ADENO. Our data underscores the importance of biomarker testing in future trials targeting TIGIT, considering age and sex disparities. Despite extensive safety profiles reported in numerous clinical trials investigating anti-TIGIT drugs and combination therapies across various tumor types, their oncological efficacy in bladder cancer remains uncertain [26].

These findings underscore the distinct expression profiles of TIGIT, LAG-3, and TIM-3 as promising immunotherapeutic targets in variant histologies of bladder cancer. In light of the limited efficacy of PD-1/PD-L1 monotherapy, there is a growing imperative to explore alternative co-inhibitory pathways. The consistent expression of TIM-3 and TIGIT across age, sex, and tumor stage suggests their potential utility as broadly applicable checkpoint targets, while the divergent expression patterns of LAG-3 point toward non-redundant, complementary roles within the immunosuppressive tumor microenvironment. Integrating these targets into rationally designed combination strategies may help overcome resistance to conventional immune checkpoint inhibitors.

In the context of personalized medicine, these insights emphasize the urgent need for robust biomarker-driven approaches to refine patient stratification and optimize therapeutic outcomes [27]. Clinical trials have demonstrated clinical feasibility of novel drugs targeting LAG-3, TIM-3, or TIGIT [28,29,30]. Combining anti-TIGIT and anti-LAG-3 with conventional anti-PD-1/anti-PD-L1 therapies has been shown to improve median survival in non-small cell lung carcinoma and melanoma [25,30]. Interestingly, an in vitro model has shown that chemotherapy increased LAG-3 expression, which makes combining novel immune checkpoint inhibitors with conventional chemotherapy promising in difficult-to-treat cancers such as VHs [11]. Furthermore, the preliminary results suggest that high TIM-3 expression correlates with treatment response, as it has been described in various cancer types for PD-L1 expression [28,31]. Further analysis has revealed that the high density of positive PD1 and LAG-3/TIGIT/TIM-3 expression was associated with poor response rates and worse oncological outcomes [32]. Accordingly, these co-inhibitory receptors correlate with the exhaustion status of CD8+ cells. Accordingly, targeting these additional immune checkpoints might increase anti-tumor activity. Focusing on VHs of bladder cancer, biomarker testing is crucial due to the high heterogeneity of tumor entities summarized by VHs. Our results, especially regarding LAG-3 expression, highlight the importance of biomarker testing. Accordingly, ADENO showed no LAG-3 expression and therefore might not be a good candidate for extended immunotherapy targeting LAG-3, in contrast to SCC, whereas TIGIT showed similar expression in both tumor entities. In the background of limited therapeutic options in patients with VHs of bladder cancer, the identification of novel treatment targets for clinical trials is crucial. Therefore, characterization of expression profiles, which might serve as prognostic biomarkers, is the first step. Recent research has focused on the genomic profiling of VHs, but treatment-relevant expression patterns do not necessarily correlate. Guégan et al. described a 25-gene signature correlating with CD8 cell exhaustion and ICI response [32]. Future clinical trials must incorporate comprehensive genomic [33] and immune profiling [34] to guide treatment selection, particularly for patients with non-urothelial bladder cancers who currently lack effective standard-of-care options [35]. Expanding the immunomodulatory landscape through the inclusion of emerging checkpoints holds promise for improving outcomes in this underserved patient population. Besides the demonstrated next-generation ICI target molecules in this study, T-cell surface marker expression has demonstrated prognostic potential [36]. Interestingly, other surface biomarkers have already shown impact on survival in VHs of bladder cancer [37] that are already used as therapeutic targets [38].

The prognostic significance of co-inhibitory receptor expression in TILs was primarily observed in TIM-3 for SCC, though interpretation is constrained by our limited cohort size and should be carefully interpreted. Therefore, larger cohort studies are needed to confirm these preliminary findings. Additionally, the intrinsic limitations of TMA-based analyses, including sampling bias and limited spatial representation of tumor heterogeneity, as well as the retrospective nature of the dataset, may influence the robustness and generalizability of the findings. Furthermore, due to the limited expression of inhibitory co-receptors, specifically LAG-3 and TIM-3, in ADENO, no multivariate analysis was performed. Larger cohort studies are warranted to allow more significant statistical analysis. This study aimed to elucidate next-generation immune checkpoints; however, receptor expression does not necessarily correlate with clinical responses to corresponding antibodies. Despite presenting the largest cohort of SCC and ADENO cases in bladder cancer research, the study’s limited patient numbers necessitate cautious result interpretation.

## 5. Conclusions

In conclusion, patients with non-urothelial neoplasms of the urinary bladder face poor prognoses, and therapeutic options remain limited despite the focus on personalized medicine. This study elucidates the immunogenic landscape of these rare bladder cancer subtypes, laying the groundwork for future trials using antibodies that are currently in clinical development to expand therapeutic options by including TIM-3, TIGIT, and LAG-3 into the immunomodulatory armamentarium.

## Figures and Tables

**Table 1:** Patient characteristics.

Characteristic	ADENO, N = 27 ^1^	SCC, N = 61 ^1^	*p*-Value ^2^
Age	71 (67, 75)	66 (58, 77)	0.2
Sex			0.088
female	8 (30%)	30 (49%)	
male	19 (70%)	31 (51%)	
T-stage			0.3
<pT3	2 (7.4%)	11 (18%)	
≥pT3	25 (93%)	50 (82%)	
TIM-3 (median)	1 (0, 2)	14 (10, 20)	<0.001
TIGIT (median)	11 (5, 15)	11 (2, 28)	0.9
LAG-3 (median)	0 (0, 0)	1 (0, 8)	<0.001
Unknown	3	2	

^1^ Median (IQR); n (%). ^2^ Wilcoxon rank sum test; Pearson’s chi-squared test; Fisher’s exact test.

## Data Availability

The dataset is available on request from the authors.

## Supplementary Materials

The following supporting information can be downloaded at: https://www.mdpi.com/article/10.3390/cancers17132210/s1, Supplementary Figure S1: Immunohistochemistry for the three co-inhibitory receptors LAG-3, TIGIT and TIM-3.
