# Supplementary material for: Expression Profiles of Co-Inhibitory Receptors in Non-Urothelial Bladder Cancer: Preclinical Evidence for the Next Generation of Immune Checkpoint Inhibitors
Source: Cancers (Basel). 2025 Jul 1;17(13):2210. doi: 10.3390/cancers17132210 (PMC12248772; doi:10.3390/cancers17132210)
Supplement: Supplementary file 1 [file cancers-17-02210-s001.zip › cancers-3621281-supplementary.pdf]

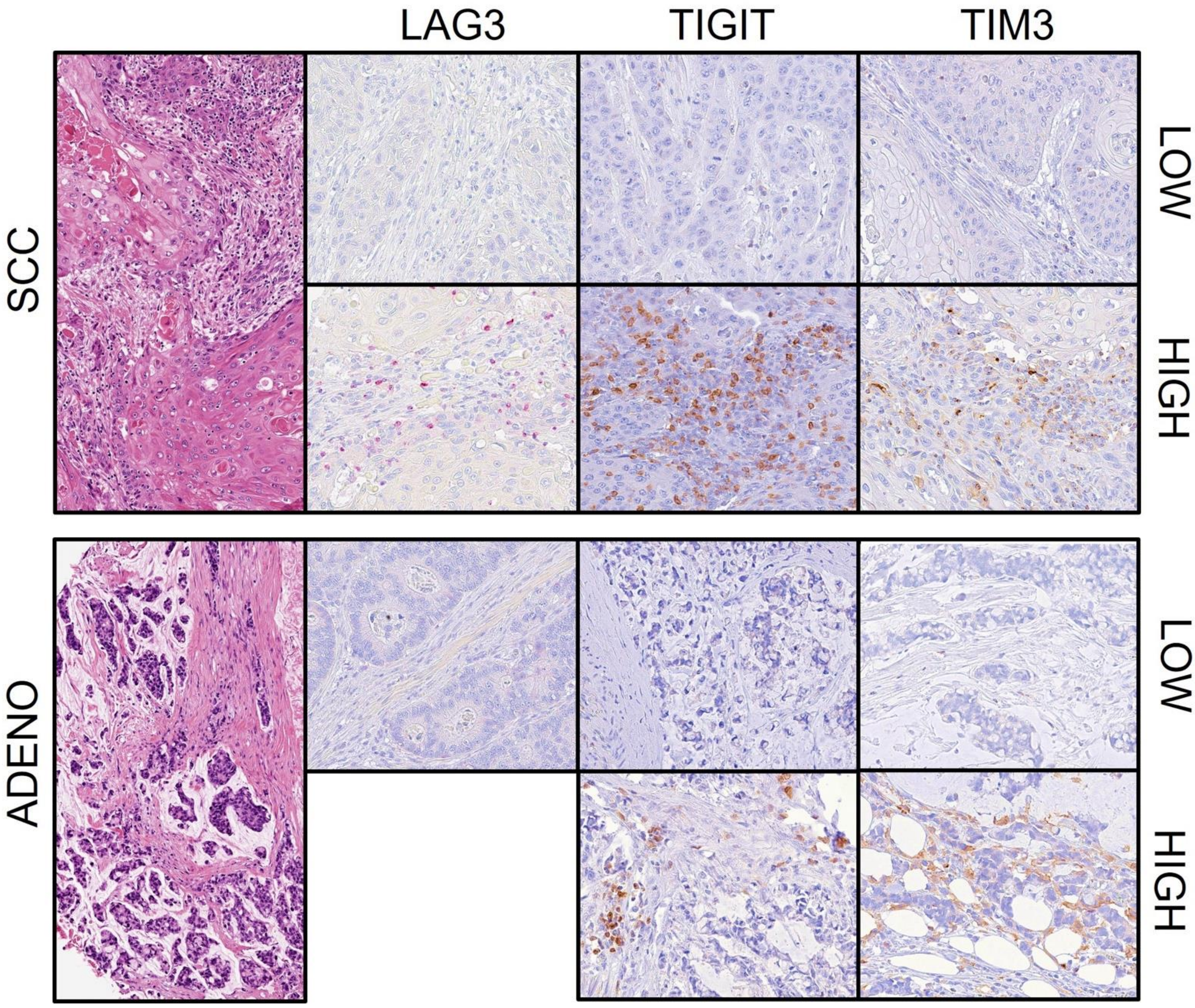

Supplemmentary Figure S1: Immunohistochemistry for the three co-inhibitory receptors LAG-3, TIGIT and TIM-3
